# Toxicity of Environmental Contaminants

**DOI:** 10.1155/2015/702439

**Published:** 2015-08-04

**Authors:** Sunil Kumar, Patrick Hettiaratchi, Nanjappa Ashwath, Petros Gikas

**Affiliations:** ^1^Solid and Hazardous Waste Management Division, CSIR-NEERI, Nehru Marg, Nagpur 440 020, India; ^2^Department of Civil Engineering, Center for Environmental Engineering Research and Education, University of Calgary, Calgary, AB, Canada T2N 1N4; ^3^Centre for Plant and Water Science, Central Queensland University, Rockhampton, QLD 4702, Australia; ^4^Department of Environmental Engineering, Technical University of Crete, 73100 Chania, Greece

Environmental toxicology is one of the most interdisciplinary subjects and it aims to study the effects of toxic chemicals on living organisms using various toxicological methods. The experiments that examine the deleterious effects of environmental pollutants on animals provide scientific basis for developing environmental standards. Results of such experiments and the techniques and practices used to minimize environmental effects of toxic chemicals will help in formulating sound environmental policies and appropriate decision-making.

The special issue addresses various topics, such as biomarkers for environmental pollution monitoring; bioaccumulation of chemicals; bioassays and biomarkers in water monitoring and biomonitoring of water bodies; ecotoxicity of emerging chemicals; biological effects of monitoring; laboratory techniques and field quantitative structure-activity relationships (QSAR); ecosystem health and environmental health risk assessment.

This special issue is a compilation of selected peer reviewed papers that range from biomarkers, bioconcentration and toxication, oxidative stress defuse pathways to cytotoxic effects of pesticides, phytoremediation of polluted soil, and so forth.

The editors believe that the results presented in these papers are valuable and will stimulate discussion on further development of potential techniques and devices for effective assessment of deleterious effects of environmental pollutants on animals and the environment.

